# Tunable Band-Stop Filters for Graphene Plasmons Based on Periodically Modulated Graphene

**DOI:** 10.1038/srep26796

**Published:** 2016-05-27

**Authors:** Bin Shi, Wei Cai, Xinzheng Zhang, Yinxiao Xiang, Yu Zhan, Juan Geng, Mengxin Ren, Jingjun Xu

**Affiliations:** 1 The MOE Key Laboratory of Weak-Light Nonlinear Photonics, TEDA Applied Physics Institute and School of Physics, Nankai University, Tianjin 300457, China; 2 Synergetic Innovation Center of Chemical Science and Engineering, Tianjin 300071, China

## Abstract

Tunable band-stop filters based on graphene with periodically modulated chemical potentials are proposed. Periodic graphene can be considered as a plasmonic crystal. Its energy band diagram is analyzed, which clearly shows a blue shift of the forbidden band with increasing chemical potential. Structural design and optimization are performed by an effective-index-based transfer matrix method, which is confirmed by numerical simulations. The center frequency of the filter can be tuned in a range from 37 to 53 THz based on the electrical tunability of graphene, while the modulation depth (−26 dB) and the bandwidth (3.1 THz) of the filter remain unchanged. Specifically, the bandwidth and modulation depth of the filters can be flexibly preset by adjusting the chemical potential ratio and the period number. The length of the filter (~750 nm) is only 1/9 of the operating wavelength in vacuum, which makes the filter a good choice for compact on-chip applications.

Graphene, a monolayer of carbon atoms arranged in a two-dimensional honeycomb lattice, has attracted much attentions in recent years for its constant light absorption^1^,^2^, high carrier mobility^3^,^4^,^5^, bipolar electrical tunability^6^ and anomalous quantum Hall effect in electrical transport^7^,^8^. It is regarded as a promising material for plasmonic devices due to its metal-like properties in the terahertz (THz) frequency range^9^. THz technology has huge potential in biosensing, communication, spectroscopy and imaging^10^. Graphene plasmons (GPs) have shown many attractive features including tight field confinement^11^ and localization^12^, long propagation length^12^, and flexible tunability^9^,^13^. Many approaches have been proposed for efficient and convenient excitation of GPs, such as tip scattering coupling^9^,^14^, grating coupling^15^, all-optical coupling^16^, tapered slabs coupling^17^ and surface acoustic coupling^18^. Plenty of graphene-based plasmonic devices with unique properties have been investigated such as detectors^19^,^20^,^21^, modulators^22^,^23^,^24^, optical polarizers^25^,^26^ and limiters^27^,^28^,^29^. All these devices serve as basic building blocks of new generation integrated optical circuits.

Plasmonic filters are essential for information processing systems and have been widely investigated^30^,^31^,^32^,^33^,^34^. Traditional metal-based filters exhibit good performance in the visible and near-infrared region^31^,^35^. However, poor confinement of metal SPPs in the THz frequency range limits their application. In contrast, GPs have high field confinement in the THz spectral range and thus graphene-based filters promise better performance. Another difference is their tunability, while the characteristic parameters of metal-based filters are difficult to adjust once they are fabricated. To address this issue, some methods have been proposed such as combining them with liquid crystals^36^ or liquid metals^37^ with tunable permittivity. But it might be easier to change the chemical potential of graphene by an external gate voltage exploiting the special energy band structure of graphene. This permits to tune the properties of GPs and the working parameters of graphene-based filters, such as the center frequency.

Band-stop filters that selectively eliminate undesired information and suppress interfering signals, play an important role in broadband communications and information storage^38^. An ideal modulation depth and a flexibly tunable working frequency would extremely promote the performance of a band-stop filter. However, the formerly proposed designs hardly satisfy these requirements. Lei Zhang *et al.* proposed a wavelength selector by coupling a graphene ribbon with a graphene disk^34^. In their design, the modulation depth could be tuned by the chemical potential of the graphene disk. However, the selected wavelength could not be tuned efficiently. Jin Tao *et al.* proposed to place a uniformly doped graphene monolayer on a silicon grating^39^. The high effective index contrast for the surface plasmon modes is generated between the graphene on silicon and air substrates. Zhen-Rong Huang *et al.* proposed a free-standing periodically-stacked graphene nanoribbon waveguide with patterned chemical potential^40^. The effective index contrast for edge plasmon modes is achieved by setting different chemical potentials of graphene at different locations. Both designs mentioned that tuning of the chemical potential could be realized by chemical doping or by bias voltage. Though the chemical potential of graphene can be changed by chemical doping, it cannot provide real-time tuning. More importantly, neither of them presented a feasible configuration to tune the graphene chemical potential by bias voltage.

In this report, we propose a type of tunable band-stop filters based on graphene monolayers with periodically modulated chemical potential that can be treated as graphene plasmonic crystals (GPCs). Investigating the energy band diagram of the GPC, one can find a blue shift of the forbidden band with increasing chemical potential. An effective-index-based transfer matrix method (EIB-TMM) is adopted to calculate the transmission and optimize the design of the GPC filter, which are in good agreement with our numerical simulations. Such a method can greatly promote the efficiency of calculations. The center frequency of the filter can be tuned from 37 to 53 THz with a stable modulation depth as high as −26 dB and a fixed bandwidth (3.1 THz) based on the electrical tunability of graphene. Bandwidth and modulation depth can be preset freely by adjusting the chemical potential ratio and the period number. The length of the filter is only 750 nm, just 1/9 of the operating wavelength in vacuum, which makes it a very good candidate for integrated plasmonic devices.

## Results

The dispersion relation of the GPs in a graphene sheet can be described as the quasi-static form^41^


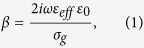


where *σ*_*g*_ is the complex surface conductivity of the graphene monolayer, *ε*_*eff*_ is the effective environment permittivity and *n*_*eff*_ = *β*/*k*_0_ is the effective refractive index for the GP mode which is inversely proportional to *σ*_*g*_.

In the THz frequency region, the complex surface conductivity *σ*_*g*_ of a graphene monolayer is described by the Kubo formulation^42^,^43^ as *σ*_*g*_ = *σ*_*intra*_ + *σ*_*inter*_, where *σ*_*intra*_ corresponds to the intraband electron-phonon scattering


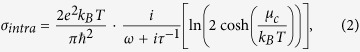


and the interband transition contribution *σ*_*inter*_ is given by


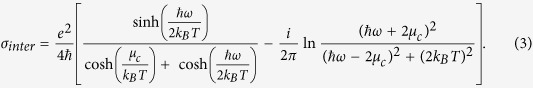


Here *e* is the elementary charge, *k*_*B*_ is Boltzmann’s constant, *T* is the ambient temperature, *ħ* is the reduced Planck constant, *ω* is the photon frequency, 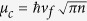 is the chemical potential where *n* and *v*_*f*_ are the charge density and the Femi velocity of the graphene respectively, and 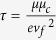 stands for the momentum relaxation time due to the charge carrier scattering. Previous reports showed that the carrier mobility *μ* of graphene on a silica substrate could reach 40000 cm^2^V^−1^s^−1^ at room temperature^4^ and that of high-quality suspended graphene^3^ could be 23000 cm^2^V^−1^s^−1^. To improve the credibility of the calculation results, 10000 cm^2^V^−1^s^−1^ is adopted as the carrier mobility in our study^44^.

**Figure 1:**
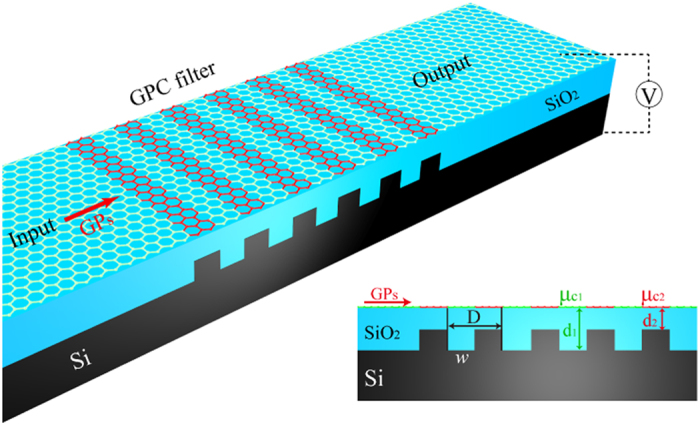
Schematic illustration of the GPC band-stop filter. A silica layer with periodic thickness is used to periodically modulate the chemical potential of graphene. The chemical potential ratio between two regions is 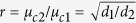 and the period is D = 2*w* = 50 nm. Influence of the silicon grating on GPs is eliminated since both *d*_1_ and *d*_2_ are always larger than 100 nm.

According to equation (1)–(3), 2, 3, *μ*_*c*_ is the only variable of *n*_*eff*_ for a specific GP mode. A graphene monolayer with a periodic *μ*_*c*_ acts as a GPC. When broadband GPs, which can be excited by tip scattering coupling^14^, propagate along the graphene monolayer, they will be periodically modulated by the GPC. A heavily doped silicon grating covered by a silica layer is used to achieve the periodic chemical potential, as shown in Fig. 1, because graphene regions with silica thicknesses *d*_1_ and *d*_2_ have different chemical potentials under a backgate bias voltage. The silica layer not only couples with the graphene but also ensures the tuning feasibility of the chemical potential of graphene. When a gate voltage is applied to the device, the charge density of the graphene is inversely proportional to the thickness of the silica layer *d* as^44^ 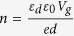, where *ε*_*d*_ is the permittivity of silica. Thus the chemical potential ratio *r* = *μ*_*c*2_/*μ*_*c*1_ between two regions is equal to 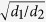. In our design, *d*_2_ is fixed at 130 nm and *r* is regulated by *d*_1_. The permittivity of silica is assumed as 3.9 and the period of the GPC is D = 2*w* = 50 nm. It is worth emphasizing that the influence of the silicon grating on the GPs could be eliminated since the thickness of the silica layer is always larger than 100 nm.

**Figure 2:**
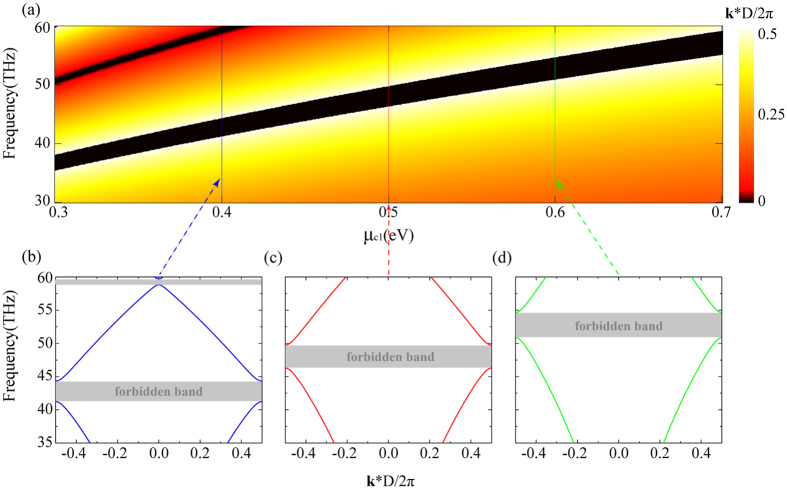
Energy band diagram of the GPC. (**a**) Energy band diagram of the GPC for different *μ*_*c*1_. **k** is the Bloch vector of the GP modes and D is the period of the GPC. (**b–d**) Energy band diagrams with *μ*_*c*1_ equal to 0.4, 0.5 and 0.6 eV, respectively. The gray regions illustrate the forbidden bands of the GPCs. In the calculations, *r* is assumed as 1.25.

By using the characteristic equation (see methods), we can calculate the forbidden band of the GPC as shown in Fig. 2(a). The chemical potential ratio is assumed as *r* = 1.25. Bloch wave vector reaches a maximum on both edges of the forbidden band and decreases as frequencies are away from the forbidden band. As shown in Fig. 2(b–d), the central frequency of the forbidden band blueshifts with increasing *μ*_*c*1_. Larger *μ*_*c*1_ causes wider bandwidths for the allowed and the forbidden bands. In particular, high-order forbidden bands appear when *μ*_*c*1_ is small enough.

The EIB-TMM is adopted to calculate the transmission and optimize the design of the GPC filters. When the GPs propagate along the graphene, reflection and transmission will occur at each interface. As an analogy of a one dimensional photonic crystal, the transfer matrix of the GPC with a period number *N* can be described as^45^,^46^


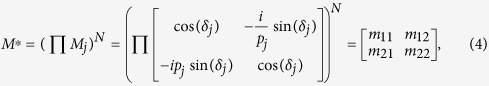


where *δ*_*j*_ = *k*_0_*n*_*eff*,*j*_*w* and 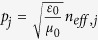 (*j* = 1, 2). The transmission of the GPs is^47^


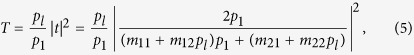


where 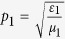 and 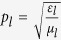 describe the external environment of the incident surface and the exit surface.

**Figure 3:**
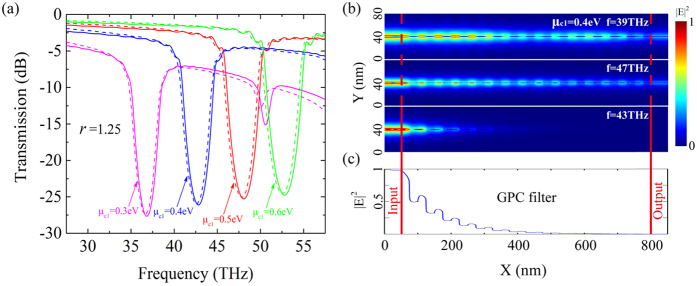
The transmission and the near-field intensity distributions of different band-stop filters. (**a**) Transmission spectra of the filters with *μ*_*c*1_ equal to 0.4, 0.5 and 0.6 eV, respectively. The solid lines are calculated by EIB-TMM and the dashed lines are simulated by COMSOL. (**b**) The side views of the near-field intensity distributions for the pass band frequencies and the center frequency of the stop band with *μ*_*c*1_ = 0.4 eV. (**c**) The energy intensity distribution curve for the center frequency of the stop band. Most of the energy is confined in the first four periods.

Transmission spectra with the same parameters as in Fig. 2 and with *N* = 15 are shown in Fig. 3(a). The calculated solid lines are in good agreement with the dashed lines that are simulated by a commercial finite-element method software (COMSOL Multiphysics). It should be noted that the EIB-TMM extremely promotes the calculation efficiency and leads to a significant reduction of calculation time. Frequencies in the forbidden band are greatly attenuated by the filter. The modulation depth for the center frequency is as high as −26 dB. The filter exhibits a quite good electrical tunability. When *μ*_*c*1_ increases from 0.3 to 0.6 eV, the center frequency experiences a blue shift from 37 to 53 THz, showing a 16 THz tunability range. Particularly, the modulation depth and the bandwidth in the tuning process remain approximately stable around −26 dB and 3.1 THz, respectively. The flexible tunability, stable modulation depth and bandwidth greatly enhance the application potential of such band-stop filters. We notice that a high-order stop band appears in the transmission spectrum when *μ*_*c*1_ is 0.3 eV, which is attributed to the high-order forbidden band of the GPC mentioned above.

Near-field intensity (|*E*|^2^) distributions (side view) at the pass band and the stop band frequencies of the filter with *μ*_*c*1_ = 0.4 eV are shown in Fig. 3(b). The band-stop filter starts at x = 50 nm, and the intensities at regions with and without the periodic chemical potential show a strong contrast. An obviously periodic reflection can be observed in the GPC region. GPs at frequencies corresponding to the pass band can easily cross over the filter since the mismatched phase prevents the formation of Bragg reflection. In contrast, the energy at the center frequency of the stop band shows negligible transmission. The periodically matched phase causes an intense energy reflection by the filter, and the energy only appears in the first four periods as shown in Fig. 3(c). It is worth emphasizing that the whole length of the filter is only 750 nm, just 1/9 of the operating wavelength in vacuum. Particularly, the ultra-short length does not sacrifice the modulation depth of the filter, which is as high as −26 dB.

**Figure 4:**
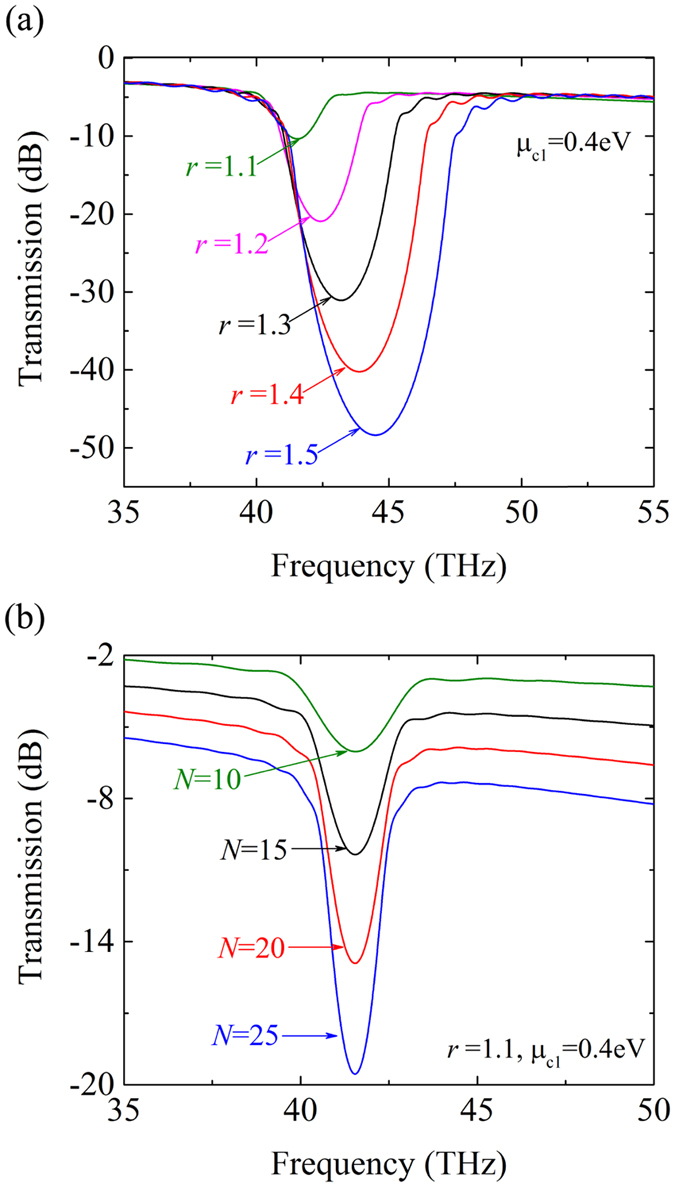
The modulation depth and the bandwidth are efficiently adjusted by the chemical potential ratio and the period number. (**a**) Transmission spectra of the filter for different *r*. *μ*_*c*1_ is fixed at 0.4 eV and *N* is equal to 15. (**b**) Transmission spectra of the filter for different *N*. *μ*_*c*1_ is fixed at 0.4 eV and *r* is assumed as 1.1.

The bandwidth and the modulation depth can also be efficiently regulated by the chemical potential ratio and the period number of the filter. As shown in Fig. 4(a), when the chemical potential ratio becomes larger, the lower cutoff frequency of the stop band is almost invariable, while the upper cutoff frequency and the modulation depth are both increasing. As mentioned above, *r* equals 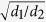 and *d*_2_ is fixed as 130 nm. Considering that 300 nm is the generally used maximum thickness of the silica layer in practical applications of graphene devices, we set the maximum *d*_1_ as 300 nm and thus the largest *r* is equal to 1.52. Therefore, *r* ranges from 1.1 to 1.5 in our simulations.

Moreover, with increasing period number the modulation depth increases while the bandwidth has a fixed value, as shown in Fig. 4(b). These results suggest that one can adjust the chemical potential ratio to reach the required bandwidth and then change the period number to achieve a suitable modulation depth. For a broad-band filter, the corresponding *r* being usually large, the modulation depth can be high enough for a relatively small *N*. For example, the modulation depth is about −25 dB with *r* = 1.4 and *N* = 10. For a narrow-band filter, more periods are required to achieve an ideal modulation depth. For example, the modulation depth is about −25 dB with *r* = 1.1 and *N* = 31.

In addition, one can see that the actual output intensity of the pass band signal decreases with increasing *N*, which is caused by the intrinsic loss of graphene after a long propagation. For a practical application, the attenuation of the signal should not be larger than a threshold value. Here, −4.3 dB is selected as the threshold, in which the output intensity is attenuated to the 1/*e* value of the input intensity. Since the transmissions at the frequencies in the pass band are not same, we select a frequency which is two bandwidths away from the center frequency (38 THz in Fig. 4(b)) as the representative of the pass band signal. As shown in Fig. 4(b), the attenuation at 38 THz equals −3.74 dB and −4.92 dB with *N* equal to 15 and 20, respectively. Further calculations show that *N* = 18 is the largest adoptable period number for *r* = 1.1 and *μ*_*c*1_ = 0.4 eV, and the corresponding attenuation is −4.18 dB.

## Discussion

In this report, a novel kind of band-stop filters based on graphene monolayers with periodic chemical potentials has been proposed. Such a graphene monolayer has a periodic *n*_*eff*_ for GP modes and can be treated as a GPC with a forbidden band that exhibits a blue shift with increasing chemical potential. EIB-TMM is adopted to efficiently calculate the transmission and optimize the design of the GPC filter. The results are in good agreement with the numerical simulations. The filter has a stable modulation depth which is as high as −26 dB and a fixed bandwidth 3.1 THz while the center frequency is flexibly tuned from 37 THz to 53 THz. Moreover, the bandwidth and the modulation depth of the filter can be freely customized by adjusting the chemical potential ratio and the period number. In particular, the length of the filter is 750 nm, which is only 1/9 of the operating wavelength in vacuum. Such a simple realization will pave the way for graphene-based plasmonic devices, such as switches, sensors and on-chip optical interconnects.

## Methods

The energy band diagram of the GPC is calculated by the following characteristic equation^24^,^48^


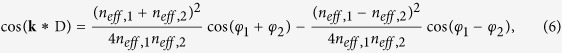


where **k** is the Bloch vector of the GP mode, *φ*_1_ = *k*_0_*n*_*eff*,1_*w* and *φ*_2_ = *k*_0_*n*_*eff*,2_*w* represent the plasmonic phase change in the graphene with the silica thickness of *d*_1_ and *d*_2_, respectively.

## Additional Information

**How to cite this article**: Shi, B. *et al.* Tunable Band-Stop Filters for Graphene Plasmons Based on Periodically Modulated Graphene. *Sci. Rep.* **6**, 26796; doi: 10.1038/srep26796 (2016).
